# Altered innate functions of myeloid dendritic cells in ANCA-associated vasculitis

**DOI:** 10.1186/1479-5876-10-S3-P20

**Published:** 2012-11-28

**Authors:** Cécile Braudeau, Antoine Néel, Marie Rimbert, Mohamed Hamidou, Régis Josien

**Affiliations:** 1 CHU Nantes, Laboratoire d’Immunologie, Nantes, France; 2 INSERM Center of Research in Transplantation and Immunology, UMR 1064, Nantes, France; 3 CHU Nantes, Service de Médecine Interne, Nantes, France; 4 Université de Nantes, Faculté de Médecine, Nantes, France

## Background

Dendritic cells (DC) are critical effectors of innate and adaptive immunity, acting both as sentinels that detect the presence of pathogens and as key antigen-presenting cells that regulate the adaptive immune response. Therefore, DC play a crucial role in the control of autoimmune responses. We previously showed that blood DC numbers were strongly reduced in ANCA-associated vasculitis (AAV) likely due to their recruitment in tissues. Here, we assessed the ex vivo responsiveness of blood DC from AAV-patients to Toll-like receptors (TLRs) stimulation.

## Materials and methods

Blood samples from 10 untreated patients with AAV during flares and before any immunosuppressive treatment (AP) were analyzed, along with 9 AAV patients in remission (RP) and 11 age-matched healthy controls (HC). Intracellular cytokine (IL-12, TNF-α, IFN-α) production by blood DC was assessed by 8-colors flow cytometry after stimulation by Toll-like receptors of whole blood samples.

## Results

We found that myeloid DC (mDC) from patients in acute phase exhibited a decreased IL-12 production after TLR3, 4 and 7/8 stimulation compared to patients in remission and healthy controls. These mDC also produced less TNF-α after TLR3 stimulation. Moreover, we observed a reduction in the frequency TNFα-producing plasmacytoid DC (pDC) upon TLR7/8 triggering in AP patients compared to RP patients and HC.

## Conclusion

Our data show that circulating mDC from patients with AAV exhibited an altered response to several TLR ligands, with a notable decrease in IL-12 production. These unexpected results suggest the innate functions of DC especially in response to pathogens are impaired during AAV.

